# Primary CNS Burkitt Lymphoma: A Case Report of a 55-Year-Old Cerebral Palsy Patient

**DOI:** 10.1155/2018/5869135

**Published:** 2018-06-24

**Authors:** Kathryn Bower, Nilay Shah

**Affiliations:** ^1^Section of Hematology/Oncology, West Virginia University, Morgantown, WV, USA; ^2^Alexander B. Osborn Hematopoietic Malignancy and Transplantation Program, West Virginia University, Morgantown, WV, USA

## Abstract

With primary central nervous system lymphoma (PCNSL) being a rare disease, the subtype of Burkitt lymphoma (BL) presenting as a sole CNS lesion is an even more exceptional diagnosis. A case of coexistent primary CNS Burkitt lymphoma (PCNSBL) with cerebral palsy (CP) is presented. A 55-year-old Caucasian male presented with increasing bilateral lower extremity weakness above his baseline in addition to signs of increased intracranial pressure. Four abnormal enhancing masses were detected on MRI with biopsy results consistent with Burkitt lymphoma. Complete staging workup was completed with no evidence of extra-CNS disease noted on PET/CT, bone marrow biopsy, or cerebral spinal fluid analysis. The patient was treated with intravenous as well as intrathecal chemotherapy and found to be in a complete remission at six months. Recurrence in the CNS was observed four months later with treatment consisting of whole brain radiation as well as intrathecal chemotherapy. Thirty months after diagnosis, the patient remains disease-free. To our knowledge, this is the first case of PCNSBL in the setting of CP. A review of literature regarding treatment options in this controversial setting is provided.

## 1. Introduction

Primary central nervous system lymphoma (PCNSL) has historically been an uncommon disease entity since it was first discovered. Recent reviews, however, indicate that cases continue to arise at increasing numbers [1–3]. Incidence rose three-fold between the years of 1973 and 1984; however, the rate of increase is currently trending toward stabilization [4]. This is perhaps due to the invention of highly active antiretroviral therapy (HAART) for acquired immunodeficiency syndrome (AIDS) as immunocompromised individuals remain at 300% increased risk for PCNSL [3], and the average age of diagnosis is 40 in the immunosuppressed population versus 55–61 years of age in those who are immunocompetent [3]. Even as the incidence of PCNSL rises, it remains a rare disease with a mere 7% incidence rate. The subset of those individuals with primary CNS Burkitt lymphoma (PCNSBL) constitutes an even scarcer population comprising just 3–5% of the PCNSL cases [1–3]. Only 36 cases of PCNSBL were found worldwide after a thorough literature review (Table 1).

With such diminutive evidence on the most effective way to treat these patients, no standard of care exists, and the chosen therapy has been anything but uniform. Many have elected various combinations of intravenous (IV) chemotherapy, intrathecal (IT) chemotherapy, and radiation therapy. A backbone of IV high-dose methotrexate (HD-MTX) proves to be the most significant prognostic variable with regard to treatment [3]. However, the optimal role for IT MTX as well as radiation has yet to be defined.

Recently, there has been doubt amongst professionals whether whole brain radiation therapy (WBRT) should be implored for these patients. In those who receive WBRT, approximately 61% relapse within the radiation field, and the risk of significant neurotoxicity, is 25–35% at 5 years with death occurring in one-third of those patients [4–6]. This toxicity proves especially detrimental in individuals greater than 60 years of age [4], with recent assertions that using WBRT in children is no longer necessary or acceptable due to significant risk of long-term neurotoxicity [7]. The German PCNSL Study Group is the largest and only phase III randomized trial comparing IV chemotherapy ± WBRT which revealed no significant difference in overall survival (OS) (44.2 versus 59.0 months, *p* = 0.78) when WBRT was added to HD-MTX-based chemotherapy in those patients with a complete response (CR) [8]. In a subset of patients who did not reach a CR, however, the addition of WBRT did show prolonged progression-free survival (PFS) (5.0 versus 2.9 months, *p* = 0.002) but did reveal a difference in OS (27.4 versus 18.2 months, *p* = 0.119) [8].

Several different therapeutic options, including IT chemotherapy and complete surgical resection, have also been questioned. As IV HD-MTX crosses the blood-brain barrier, many believe IT only increases toxicity with little to no additional benefit. Complete resection of the tumor has also been challenged in the past as it potentially increases neurologic deficits without any survival benefit [5]. Recently, the German PCNSL Study Group-1 (GPSG-1) trial has refuted this, stating there may be significant PFS [2]. Discrepancies may be attributed to the advances that have been made in neurosurgical techniques over the last decade [2].

Patients with PCNSL have an extremely poor prognosis, with an OS of approximately 12–18 months [1, 3, 9]. Without treatment, this number dwindles to 1.5–3.3 months [10]. Therefore, it is imperative that this disease be treated with the best available option to improve the expected survival of these individuals. Here, we present a case of an adult HIV-negative male with PCNSBL in the setting of cerebral palsy (CP) and our approach to treatment with long-term follow-up.

## 2. Case

**Figure 1:**
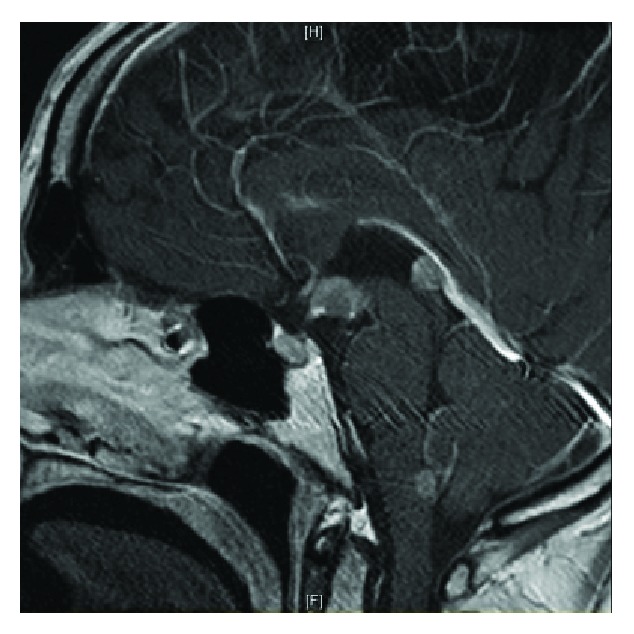
MRI brain, T1 sagittal + gadolinium, demonstrated lesions within hypothalamus, pineal gland, trigon of the right lateral ventricle, and foramen of Magendie at diagnosis.

A 55-year-old Caucasian male with past medical history of cerebral palsy (CP) presented with nausea, vomiting, thirty-pound weight loss, and worsening bilateral lower extremity weakness for one month. A computerized tomography (CT) angiogram of the brain revealed a suprasellar mass facilitating transfer to our institution for further management. Magnetic resonance imaging (MRI) of the brain indicated abnormal enhancement along the ependymal margin of the frontal horns of the bilateral lateral ventricles with four distinct abnormal enhancing mass lesions in the hypothalamus (11 × 12 × 13 mm), pineal gland (8 × 8 × 9 mm), the trigon of the right lateral ventricle (5 × 5 × 4 mm), and the foramen of Magendie (7 × 6 × 9 mm) which demonstrated restriction diffusion indicating hypercellularity (Figure 1).

An endoscopic biopsy of the third ventricle floor lesion was performed with pathology revealing sheets of intermediate size monotonous lymphoid cells displaying high nuclear-to-cytoplasmic ratio with dispersed chromatin and indistinct nucleoli. Numerous apoptotic cells and mitotic figures with foci of necrosis were observed. The tumor cells displayed CD 20 with coexpression of CD 10 and were negative for BCL 2, BCL 6, CD 3, and CD 5. EBER in situ hybridization was also negative. Fluorescent in situ hybridization was positive for [11, 12] (MYC/IHG) fusion in 97% of the cells and loss of BCL2 in 96%. These results appeared to be consistent with Burkitt lymphoma.

Staging workup was obtained which only revealed concern for extra cranial disease present at T12-L1 and L2-L3 consistent with subarachnoid nodular pial metastases on MRI of the lumbosacral spine. PET/CT disclosed no evidence of extra-CNS disease. A lumbar puncture and bone marrow biopsy were performed and found to be negative for disease. In the absence of extra-CNS disease, the patient was diagnosed with PCNSBL.

**Figure 2:**
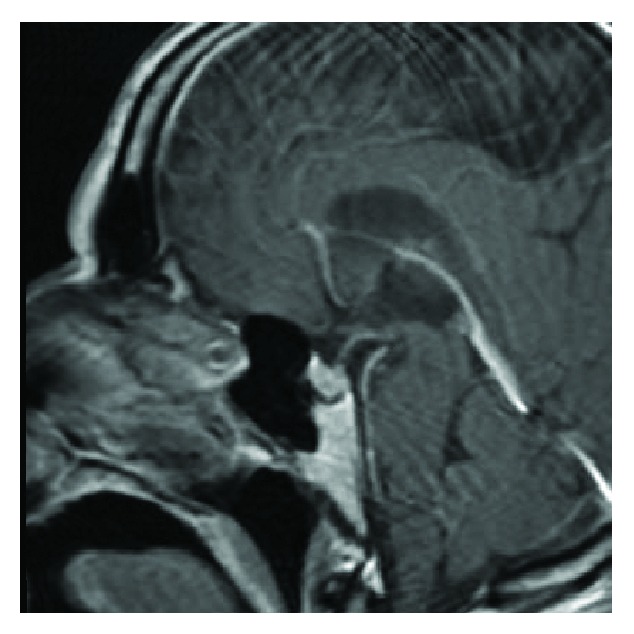
MRI brain, T1 sagittal + gadolinium, revealing complete resolution of all four mass lesions after receiving IV and IT chemotherapy.

Patient was started on IV HD-MTX (3.5 grams per meter squared) and cytarabine (2 grams per meter squared) per Ferreri regimen [13] with the addition of IT MTX/cytarabine every 21 days for four cycles. Dose was reduced by 25% for cycle three due to persistent cytopenias and toxicity including renal dysfunction with delayed MTX clearance. A repeat brain MRI was obtained after 6 months which indicated complete remission with no evidence of disease (Figure 2).

Four months later, the patient began having generalized weakness with visual disturbances and headaches. A repeat brain MRI at that time revealed interval development of markedly abnormal signal in the pons extending to the midbrain and dorsal medulla. Repeat cerebral spinal fluid (CSF) analysis showed rare atypical lymphocytes consistent with relapse of lymphoma. He underwent WBRT, receiving 30 Gray (Gy) over 17 fractions with an additional boost to the midbrain lesion with 9 Gy in 8 fractions. Currently, he is disease-free at 30 months s/p diagnosis.

## 3. Discussion

To our knowledge, our patient is the only PCNSBL case with a past medical history of CP. No literature was discovered that revealed a connection between these two diseases, and further research may be warranted if similar cases develop in the future. It would have been easy to dismiss the presenting symptoms as part of his CP; therefore, caution must be taken to not be blinded by a patient's past medical history in attempting to decipher the etiology of new symptoms. Primary CNS lymphoma should remain a consideration for those presenting with neurological symptoms regardless of their history.

The optimal treatment for PCNSBL continues to elude us, and no standard of care exists at this time. The paucity of cases provides little opportunity for randomized controlled trials; therefore, clinicians have been forced to extrapolate based upon recommendations for Burkitt lymphoma residing outside as well as PCNSL regimens. Many of the reported cases of PCNSBL used HD-MTX +/−, some variation of cyclophosphamide, doxorubicin, vincristine, and prednisone (CHOP) therapy. A few studies attempted additional agents such as ifosfamide and procarbazine, but had little success. Several used IT MTX alone or in combination with cytarabine and steroids.

Our treatment decision was based upon the Ferreri regimen, which is the only randomized trial for PCNSL [13]. Since our patient suffered from the added rarity of Burkitt lymphoma subtype, the addition of IT chemotherapy was made based on standard practice for extra-CNS Burkitt lymphoma as it has a high incidence of CNS penetration. While there has been some debate regarding the need for IT chemotherapy in addition to IV HD-MTX, these patients were mostly non-Burkitt type (diffuse large B cell) PCNSL. Even with the baseline cognitive deficits of our patient, he was able to tolerate an aggressive chemotherapy regimen that included IT and eventually WBRT without any permanent neurological complications to date. Although he did develop some mild delirium while hospitalized for WBRT and IT MTX after relapse, this promptly resolved prior to discharge. The decision at that time was made to withhold any further IT therapy as no further evidence of disease was present and the risk of toxicities outweighed the current benefits. He remains disease-free at 30 months.

The role of IT therapy has been challenged in the face of HD-MTX therapy being able to cross the BBB. Ferreri et al. have shown that there is no survival benefit when using IT in addition to HD-MTX with regard to PCNSL of all types, with 2-year OS rates of 51 ± 5% with IT versus 50 ± 6% without IT [14]. There are also reports of increased neurotoxicity when adding concurrent IT to HD-MTX with no survival benefit [8]. Neither of these studies separated those with Burkitt subtype, however. Therefore, our decision for IT administration was based upon extrapolation from accepted therapy for extra-CNS Burkitt lymphoma being IT MTX/Ara-C in addition to systemic chemotherapy.

We present a case of relapsed lymphoma that responded to WBRT of 30 Gray in 17 fractions with boost of 9 Gray in 8 fractions which was tolerated well with a complete response (CR), and no evidence of disease at 18 months after radiation was completed. Many studies have attempted to investigate the benefits and toxicities associated with the use of WBRT, which challenges the prior approach to this entity. There seems to be a trend away from WBRT, especially in those individuals > 60 years of age and children with no evidence of residual disease after IV chemotherapy, as long-term neurocognitive consequences have been found to outweigh the benefit gained by these patients with neurotoxicity being fatal even without evidence of recurrent disease [4].

Recent studies have shown no OS benefit when adding upfront WBRT to HD-MTX-containing regimens. Ferreri et al. have shown an OS of 25 ± 4% at 2 years with WBRT alone which was significantly inferior to both MTX-containing chemotherapy as well as MTX combined with WBRT, revealing a 2-year OS of 34 ± 10% and 45 ± 3%, respectively [14]. This OS difference between WBRT with MTX versus MTX alone was not significant; therefore, it is suggested that those individuals at a high risk of neurotoxicity forego WBRT unless relapse or refractory disease is apparent. We therefore chose to forego WBRT in the initial setting, reserving it for relapsed disease in our patient. Debate also exists regarding the optimal dose of WBRT, as each study utilized different fractions and dosages. Hyperfractionation has also been noted to have increase toxicity as compared to standard dosage with neurotoxicity rates of 23% and 3.7%, respectively [5]. This has been shown to be most prevalent in those receiving > 50 Gray [9].

The concept of complete surgical resection has fallen under scrutiny in recent years as well. Previously, many subscribed to complete resection of the mass with recent reports challenging this citing an increase in postoperative neurological deficits with no OS benefit [2, 3, 15]. Currently, the decision for excision when CNS lesions are the only areas of disease is made on a case by case basis with regard to tumor location and expected postsurgical deficits.

Despite adequate initial treatment with chemotherapy with or without WBRT, approximately 40–50% of PCNSL patients relapse within the first 5 years [3]. This obviates the need for obtaining better treatment modalities, not only first line but also for relapsed or refractory disease. The median post relapse survival rate approximates 2 months with a 2-year OS of 8% [3]. Our patient is now 18 months status post a second CR. A few small studies have evaluated the role of bone marrow transplant as a potential treatment for PCNSL in the relapsed and refractory setting as these individuals have a significantly increased risk of death [2, 16, 17]. Randomized phase II trials must be undertaken to thoroughly evaluate this option with regard to such a specific disease population.

Our patient subscribed to the current statistics of recurrence of disease within the first five years despite a complete response to initial treatment. He is currently disease-free for 18 months after reinduction with WBRT, which is longer than the average survival of such individuals, and remains without any significant neurotoxicity. Using our approach of IV and IT chemotherapy in PCNSBL upfront and reserving WBRT for the relapsed setting, our patient has far exceeded the median post relapse survival rate. This suggests a potential benefit to our approach. Regardless of the relatively favorable outcome of our patient, it remains that clear optimal treatment continues to be elusive. Substantial advances are required with regard to PCNSBL, as it remains a significant challenge to patients and physicians alike.

## Figures and Tables

**Table 1:** Reported PCNSBL cases. Cy: cyclophosphamide; OS: overall survival; WBRT: whole brain radiation therapy; CHOP: cyclophosphamide, doxorubicin, vincristine, and prednisone; Dex: dexamethasone; IVIG: intravenous immunoglobulin; MTX: methotrexate.

Author	Year	Age/sex	How it is diagnosed (LP versus mass)	Treatment and OS
Gawish [18]	1976	8/M	Left frontoparietal mass extending across midline and through the skull	Complete resection with recurrence. Subtotal resection with Cy. OS of 3 years
Valsamis et al. [19]	1976	6 m/M	Left parietal, bilateral temporal, and post pituitary mass with abdominal and periaortic nodal involvement	Resection, steroids, WBRT, and spinal irradiation with recurrence, IT MTX. OS of 23 months
Tanaka et al. [20]	1977	49/M	Right thalamus to midbrain mass	Subtotal resection. OS of 4.5 years
Tanaka et al. [20]	1977	58/M	Right temporal mass	Subtotal resection. Recurrence. OS 3 months
Tanaka et al. [20]	1977	42/M	Left deep parietal to occipital mass	Pred with partial resection. Recurrence. Vincristine and Cy with radiation. Vincristine, bleomycin, Cy, steroids. OS of 2.5 years
Giromini et al. [11]	1981	11/M	Left temporooccipital mass	Complete resection
Hegedüs [21]	1984	50/F	Right lower parietal lobe mass	Post mortem finding
Kobayashi [22]	1984	55/F	Right temporoparietal mass	Complete resection. Recurrence with reresection. OS of 2 months
Pui et al. [23]	1985	6/M	T2-5 mass	Laminectomy and CHOP (without prednisone). OS > 2 years
Pui et al. [23]	1985	7/M	C7-T4 mass	Laminectomy, radiation, dex, and Cy. Recurrence. OS of 5 months
Pui et al. [23]	1985	12/M	T7-10 mass	Laminectomy, CHOP (substituting dex for prednisone). OS of 4 months
Mizugami et al. [24]	1987	6/M	T10 mass	Near complete resection, radiation, and chemotherapy. Leukemic transformation then CSF recurrence. IT MTX and cranial irradiation. OS of 20 months
Mizugami et al. [24]	1987	5/M	Epidural T12-L4 mass	Near complete resection, radiation, and chemotherapy with recurrence. OS of 7 months
Mizugami et al. [24]	1987	7/F	T11 mass	Near complete resection. Spinal radiation and chemotherapy with progression of disease. OS of 3 months
Shigemori et al. [25]	1991	49/F	Left frontal lobe mass	Resection, radiation, CHOP, and IT MTX. OS of >6 months
Tekkök et al. [12]	1991	5/M	Parasellar mass, extending to bilateral sphenoids and sella turcica	Partial resection, craniospinal radiation, CHOP, and IT MTX/cytarabine/prednisone. OS > 18 months
Toren et al. [26]	1994	6/F	CSF	Steroids, IVIG, doxorubicin, vincristine, HD MTX, with IT MTX, cytarabine, and hydrocortisone. Changed to CHOP with MTX and IT MTX, cytarabine, hydrocortisone. OS of >2 years
Mora and Wollner [7]	1999	18/M	T11 mass	Laminectomy with CHOP substitute daunorubicin for doxorubicin and radiation. Relapse and refused further treatment. OS > 8 months
Mora and Wollner [7]	1999	9/M	Epidural T9-11 mass	Laminectomy, dex, radiation, and CHOP (substituting daunorubicin for doxorubicin). Recurrence and given chemotherapy via LSA3 protocol. Second recurrence, received palliative radiation. OS > 1 year
Spath-Schwalbe et al. [27]	1999	40/M	Cerebellum and pons masses	MTX and WBRT. OS > 1 year
Wilkening et al. [28]	2001	43/F	L2-3 epidural tumor involving the dura and cauda equina	Complete resection, radiation, IT MTX, and MTX with ifosfamide and CHOP (with dex substituted for prednisone). OS of >2 years
Monabati et al. [29]	2002	49/F	Right parietal mass	Complete resection, CHOP, and craniospinal radiation. Refused further treatment. OS of >6 months
Daley et al. [30]	2003	13/F	L1-2 epidural mass	Complete excision, CHOP with MTX, and IT MTX and cytarabine and steroids. OS of >5 years
Shehu [31]	2003	8/M	Left temporal and right orbit masses	Cy, vincristine, and MTX with IT cytosine arabinoside. OS of 11 months
Abel et al. [32]	2006	50/M	Central and right thalamus mass	Unknown
Gobbato et al. [15]	2006	38/M	Right frontotemporoparietal subdural mass	Craniotomy. OS of 11 days
Kozáková et al. [33]	2008	60/F	Sellar/pituitary mass	Complete resection
Gu et al. [34]	2010	75/F	Third and left lateral ventricle masses	WBRT. OS of >9 months
Takasu et al. [35]	2010	71/M	Hypothalamus and third ventricle mass	Partial resection and WBRT
Jiang et al. [36]	2011	14/M	Right lateral ventricle mass	Complete resection, radiation, and MTX, vincristine, predisone, and leucovorin. OS of >18 months
Lim et al. [10]	2011	43/F	Medulla oblongata mass, CSF involvement	MTX, vincristine, and procarbazine with IT MTX and WBRT. OS of 7 months
Akhaddar et al. [37]	2012	13/F	Right infratemporal and cavernous/maxillary/sphenoethmoidal sinus mass	Chemotherapy
Jiang et al. [38]	2012	69/M	Right temporal and occipital lobe, cervical spine, and cauda equina masses; CSF involvement	DLBCL/BL subtype. WBRT, spinal radiation with recurrence. HD MTX and cytarabine with rituximab
Yoon et al. [39]	2012	10/M	Suprasellar, cerebellum, and 3rd ventricle masses; CSF involvement	HD MTX and cytarabine with IT cytarabine, MTX, and hydrocortisone. OS of >7 years
Yoon et al. [39]	2012	32 m/M	Sellar mass extending to orbit/sphenoid, CSF involvement	HD MTX and cytarabine with IT cytarabine, MTX, and hydrocortisone. Relapse, treated with IT cytarabine, MTX, and hydrocortisone with WBRT and spinal radiation. Then received prednisone, vincristine, and cyclophosphamide with IT. OS of 9 months
Alabdulsalam et al. [40]	2014	18/M	4th ventricle mass	Craniotomy with HD MTX with rituximab-CHOP and IT MTX, cytarabine, and hydrocortisone. OS of >18 months
